# Why has low fertility become a polarized issue?

**DOI:** 10.1007/s11186-026-09760-5

**Published:** 2026-10-23

**Authors:** John Iceland

**Affiliations:** Department of Sociology, Penn State University, 601 Susan Welch Liberal Arts Building, University Park, PA 16802 USA

**Keywords:** Fertility decline, Low fertility, Depopulation, Moral foundations theory, Policy, Historical demography

## Abstract

Most countries today have below-replacement fertility and the global population is projected to decline before the century’s end. Potential consequences include fiscal challenges of an aging population, slower economic growth, and weakened kinship networks. Yet the policy response of demographers has been relatively muted compared to their more active role in promoting population control during periods of rapid growth. While substantive considerations help explain disagreement over whether low fertility is a problem, this paper examines how competing moral frameworks—rooted in Social Justice and Social Order—may also shape public and academic responses to low fertility. I begin with a historical comparison between the politics of population growth and today’s debate about population decline and develop a framework linking moral orientations to different understandings of demographic change and appropriate policy responses. I then use large language models to analyze the moral framing of fertility research published in eight leading demographic journals from 1947 to 2025. While most article abstracts contained neither moral frame, among those that did, 83% employed a Social Justice frame and 17% a Social Order frame, with Social Justice framing becoming increasingly prominent over time. I conclude by arguing that the predominance of Social Justice orientations may contribute to the muted response to low fertility, given that efforts to increase fertility align more closely with Social Order concerns, and that recognizing the moral dimensions of demographic debates can facilitate more constructive engagement.

## Introduction

Current projections point to profound demographic changes driven by sustained low fertility. South Korea’s population, for instance, is projected to more than halve by the century’s end (United Nations, 2024). There are several potential negative effects of population decline, including an aging population and a strained safety net, lower levels of economic growth, and smaller kinship support networks. Given these risks, one might expect demographers to play a leading role in drawing attention to this unprecedented change and offering policy responses. Yet, compared to the prominent role that demographers played in promoting fertility control in the earlier period, the response to low fertility has been relatively muted. This in part reflects substantive disagreements about whether low fertility will have the negative impact that some predict. However, one contributing reason explored in this study is that that low fertility has become a politically polarizing issue. Progressives tend to downplay it, while conservatives often argue that it is a pressing problem (Evans, 2024; Haspel, 2025).

Providing a historical perspective to past demographic challenges helps clarify the current debate. During the period of rapid population growth in the 20th century, demographers and the public largely rallied around the effort to reduce fertility. The motivations underlying this effort varied over time and place, ranging from early support from eugenicists to reduce population growth among poor people and countries, to a later concern among environments with overcrowding and resource depletion (Caldwell & Caldwell, 1986; Connelly, 2008; Hodgson, 1988; Merchant, 2017, 2021). By the 1990s, a consensus emerged that not only did rapid population growth pose a threat to sustainability, fertility decline was in fact necessary to advance reproductive health and the empowerment of women, as codified at the International Conference on Population and Development held in Cairo in September 1994 (Cohen & Richards, 1994; McIntosh & Finkle, 1995).

With persistent declines in global fertility, the global population is currently expected to peak in the 2080s (United Nations, 2024). Moreover, there is a growing concern with below-replacement fertility in many countries (typically defined as a total fertility rate (TFR) below 2.1), and the potential for steep demographic decline in places with especially low fertility rates, such as in East Asia and Eastern Europe. Nonetheless, there is considerable debate about the pace and effects of these demographic trends. Some argue that fertility decline is an existential crisis while others see it as a relatively minor challenge that can reasonably be managed (Bloom et al., 2025; Root et al., 2025; Teitelbaum & Winter, 1985).

Just as people with a variety of motivations supported policies to control population growth, commentators who promote higher fertility do so for different reasons. Some have pragmatic concerns, such as the challenge of supporting an aging population or economic stagnation that might accompany demographic decline (Jones, 2022; Peters, 2022; Spears & Geruso, 2025). However, since many of those in the public sphere who promote higher fertility are politically conservative (Bloom et al., 2025), some have expressed concern that pro-natalism is subterfuge for strengthening traditional family values that disempower women, as the burden of childbirth and childrearing typically fall on women and is associated with the gender earnings gap (Blau & Kahn, 2017; Glauber, 2018; Goldin, 2021; Goldin et al., 2017; Iceland & Redstone, 2020). Similarly, it could be said that *opposition* to pronatalism is likewise grounded in moral concerns, especially with regards to the implications of pronatalism for women’s empowerment.

This paper develops a conceptual framework for understanding why low fertility has become polarized. I argue that these disagreements can, in part, be understood as reflecting two broad moral orientations—Social Justice and Social Order—that shape how individuals interpret demographic change. This study goes beyond identifying that people have different views based on their political orientation, which is well-known, by explaining *why* they differ. In this paper I explore how these moral frameworks have influenced public and academic responses to population decline, including among demographers, who are not immune to the tensions that are in play among the public. I focus largely on the U.S. context, though these moral frameworks are likely useful for understanding differences in opinion on this issue globally. I begin with a historical comparison between the politics of population growth and today’s debate about population decline. I then describe how moral worldviews shape interpretations of fertility trends and why this might contribute to demographers being cautious about engaging with the potential challenges of low fertility. I follow this with an empirical analysis of demographic research that confirms that fertility issue are more commonly discussed from a Social Justice frame than a Social Order one, and that the disparity in framing has increased since the 1970s. I end with a discussion of the main approaches for addressing low fertility based on the different moral worldviews and how these views can influence disagreements on this issue.

## Historical background

The Population Association of America (PAA) was founded in the early 1930s and the first meeting included both activists and scholars (Weeks, 1996). The activists included advocates for immigration restriction, birth control proponents, and biological Malthusians concerned with high birth rates among the lower classes. The founding occurred near the peak of the eugenics movement, which sought to improve the “quality” of the population by encouraging reproduction among those deemed fit (e.g., upper class old stock Americans) and discouraging it among others (Hodgson, 1991; Merchant & Alexander, 2022). Although activists played an important initial role in the PAA, population scientists soon dominated the new organization, as the eugenics movement began losing credibility with the rise of Nazism in the 1930s (Hodgson, 1991).

Nonetheless, population control efforts gained ground in the post-World War II period, at a time of continued high fertility and population growth in developing countries. Governments, philanthropic foundations, and international organizations such as the World Bank and United Nations supported and invested heavily in family planning programs, often with strong support from the demographic community (Greenhalgh, 1996; Merchant, 2017, 2021; Ramsden, 2003). These efforts were justified on the grounds that lowering fertility was necessary for economic growth and modernization (Bongaarts & Sinding, 2011; Merchant & Alexander, 2022; Tsui, 2001; United Nations, 1974).

Policy efforts to reduce fertility typically emphasized voluntary measures, such as family planning programs that promoted a small family ideal, educated women about how they could effectively control their fertility, and provided free or reduced-price contraception, which was especially important in low income nations (Caldwell et al., 2002; Tsui, 2001).

Over time, some advocates for fertility decline argued for more sweeping measures. Paul Ehrlich, in his influential book, *The Population Bomb* (1968: 135–136) argued that only compulsory birth regulation would achieve the necessary level of population control, and he mused that adding temporary sterilants to the water supply could prove useful. In advocating for zero population growth, Kingsly Davis (1973: 21) also argued for more coercive measures. He asked, “Why not simply limit each couple to two births, with sufficient penalties to discourage three?” He answered his own query by arguing that the “Population Establishment” would not allow it because it would be considered “compulsion.” He recognized the tradeoffs between reproductive freedom and population control, but argued that the latter was serious enough that stronger measures were needed.

While coercive approaches were for a time used in countries such as China and India, these approaches by-and-large did not receive wide support among nongovernmental organizations, demographers (as noted by Davis), or the public (Bongaarts & Sinding, 2011; Cleland et al., 2006). The movement against coercion was also influenced by the spread of human rights norms, such as those initially institutionalized by organizations like the United Nations, with its Universal Declaration of Human Rights in 1948 (Moyn, 2010; United Nations, 1948). These norms were further shaped by liberation movements of the 1950s to 1970s, which included the Civil Rights movement in the U.S., decolonization movements in newly independent countries, and the feminist movement, which reframed rights around reproductive autonomy and bodily integrity. These movements led some to attack population control as “something that white, wealthy elites—especially in the United States—perpetuated on the rest of the world.” (Connelly, 2008: 14).

By the early 1990s, population control efforts more generally were on the decline for several reasons, including the achievement of long-term replacement level fertility in much of East and Southeast Asia (and declining fertility elsewhere) and a decline in international donor funding. Perhaps even more notably, codifying the growing unease many felt with traditional population control measures, the 1994 International Conference on Population and Development held in Cairo called for a significant change in aims from just fertility reduction toward reproductive health and the improvement of the situation of women (Caldwell et al., 2002).

The shift toward rights-based, voluntary family planning in the late 20th century reflected a broader rise in human rights norms (Connelly, 2008; Moyn, 2010; United Nations, 1995). The excesses of coercive family planning in some places and the focus of population control efforts in developing (and, often, previously colonized countries) at least partially discredited population control as a moral cause. Demographers adapted by adopting the language of choice, rights, and empowerment.

This approach is consonant with a Social Justice framing of the issue of population change (discussed in more detail below). Rather than emphasizing the importance of restoring demographic equilibrium to sustain broad economic prosperity, as in mid-twentieth-century population control efforts, the focus shifted to protecting vulnerable and historically marginalized groups, particularly women, from harm. In this framing, population control became not merely a strategy to modernize or spur growth, but a way to challenge and transform an existing, patriarchal social order.

But now that most countries have achieved sustained fertility declines, and depopulation rather than overpopulation looms as a problem to be addressed in many of them, such as in Japan (TFR 1.2), Romania (TFR 1.3), Italy (TFR 1.2), and Thailand (TFR 1.0) (Population Reference Bureau, 2026), demographers lack the moral framing or language to address it. If fertility decline has long been seen as a sign of progress, and aligned with human rights values, how are demographers to advocate for fertility promotion?

## The challenge of low fertility

The global fertility rate has dropped from just under 5 in 1950 to just over two in 2025. It is now low or falling everywhere, including in low-income countries and those with religious populations. About two thirds of the world live in a nation with a fertility rate below two, including the three most populous countries—India, China, and the United States—plus the European Union. Current low levels of fertility do not simply reflect a low period fertility rate, which can be temporarily low if women are delaying childbearing and having children at older ages—it shows up in completed cohort fertility as well (Geruso & Spears, 2026).

In 2024, the U.N. projected that the global population, which was 8.2 billion in that year, would peak at 10.3 billion in 2082 and then decline to 10.2 billion by 2100. Notably, however, the 2024 projections continued a trend of population downgrades in projections since 2015, mainly due to faster-than-expected declines in fertility (Australian Government, Centre for Population, 2025; Evans, 2024; Fernandez-Villaverde, 2024; United Nations, 2024). Other studies project an earlier date for population decline. For instance, Vollset et al. (2020) project a peak of 9.7 billion in 2064, falling to 8.8 billion by 2100. Moreover, while the global population will likely not decline for several decades, the populations of many countries are already dropping—reflective of the fact that their fertility has been below replacement level for decades—including Japan, Italy, Uruguay, and Germany (World Population Review, 2026).

It is also important to recognize that persistent below-replacement fertility eventually leads to *exponential* population decline, just as above-replacement fertility led to exponential growth. That’s why, as noted at this paper’s outset, South Korea’s population is projected to more than halve by the century’s end. There is also no specific reason to believe that below-replacement fertility will rebound, given that long-term fertility declines are founded on social and economic changes now occurring nearly everywhere, such as rising living standards, increasing levels of education, and women’s empowerment (Kearney & Levine, 2025).

But is population decline associated with sustained low fertility really a problem? On the one hand, population decline has some benefits. It will make it easier to sustain natural resources and feed the global population, it will reduce emissions, and it will put less pressure on building infrastructure and housing (Smith, 2026). However, all these problems can be addressed even as populations increase. For instance, the Green Revolution made food more plentiful, disproving neo-Malthusian predictions that starvation and disease were imminent (Ehrlich, 1968; Evenson & Gollin, 2003; Meadows et al., 1972). Emissions and pollution have declined in the United States and the European Union due to the development of clean energy alternatives even as their populations and economies grew (Spears & Geruso, 2025).

On the other hand, there are reasons to believe that, at minimum, depopulation will raise several challenges. First, it could reduce future increases in standards of living. Population decline means fewer people contributing to the ideas, creativity, experimentation, and technological developments that benefit everyone. There is a generally positive correlation between population size and productivity, as larger markets produce greater innovation and benefits via specialization, trade, and scale (Peters, 2022; Jones, 2005, 2022; Mino & Sasaki, 2023). Depopulation also reduces consumption and aggregate demand, and population aging, an outcome of sustained low fertility and low mortality, is strongly correlated reductions in economic growth and less innovation (Maestas et al., 2016, 2023). Agglomeration effects of an increasing population may be replaced by de-agglomeration effects of a declining one, where supply chains fragment and local markets shrink, leading to the closure of more businesses (Sustrik, 2026).

An aging population resulting from low fertility and low mortality also increases the dependency ratio, meaning that the economic burden of supporting elderly populations will be shared by a shrinking number of people. This will put pressure on safety net programs such as Social Security and Medicare, and their equivalents in other countries. This may result in higher taxes, more eldercare, and longer working lives, and ultimately test the sustainability of generous welfare states. People may need to be more thrifty to save for their own retirements (Santa, 2026; Smith, 2026).

Low fertility also results in smaller kin groups and kin support. This is important because close kin provide many types of support as people age, affecting their health, financial health, and happiness (Verdery & Margolis, 2017). Coupled with population aging, this will increase the burden of caring for elderly parents on a smaller number of living children (Tomassini & Wolf, 2000). Older people in places with lower fertility tend to have higher levels of solitary living, lower levels of co-residence with children, and lower levels of support (Glaser et al., 2006).

Finally, at the individual-level, low fertility is a problem if it indicates a mismatch between fertility desires and outcomes. Research indicates that women in all European countries and the United States generally have, on average, fewer children than they intend, and a higher rate of unintended childlessness. The gap is largest among highly education women in most of these countries (Beaujouan & Berghammer, 2019; Berrington & Pattaro, 2014; Harknett & Hartnett, 2014; Lazzari & Beaujouan, 2026). Fertility gaps are also found Canada, Australia, and some Asian countries such as Japan and South Korea. Even as women have more children than they intend in some African countries, they have fewer than intended in others (Stone, 2019).

It is important to note that many observers are unconcerned, or only moderately concerned, with low fertility for reasons that are unconnected with moral orientations (the focus of this study). Among these, population projections are unreliable. Fertility rates change over time in unpredictable ways, and there is a possibility that fertility could rebound (Root et al., 2025). Somewhat related to the fallibility of population projections, the time horizon when population decline will occur in most places is still long. Few societies to date have experienced sustained population declines, and many societies experiencing fertility decline are still enjoying the demographic dividend of having large working-age populations and few child dependents. That said, while population projections are indeed often wrong, most recent projections have erred by underestimating the pace and magnitude of fertility decline. Reversing this trend would likely require substantial changes in the social, economic, and cultural conditions that have driven it.

There is also no consensus on the “optimal” level of fertility. Replacement fertility (i.e., a TFR or 2.1) is often used as a normative threshold, as it produces a stationary population over the long run. However, this does not mean that it is the level at which economic or social welfare is maximized (Weil, 2024, 2026). People also employ different benchmarks on what is optimal, such as maximizing economic, environmental, fiscal, or individual-fertility-preference goals, reflecting not just disputes about the effects of demographic change, but also normative disagreements about how to maximize wellbeing. This, as I discuss in more detail below, is consonant with my own argument about how moral mindsets shape not only if people see low fertility as a problem but the appropriate welfare-maximizing approaches to addressing it.

Demographers may also be less concerned with population decline because many of the potential problems it creates are economic in nature, such as whether it will slow economic growth or exacerbate fiscal challenges, and most demographers are not economists. Indeed, some of those who emphasized the potential benefits when rapid population growth was a subject of broad concern were economists making economic arguments (e.g., Jones, 2019; Simon, 1996), just as some of those raising concerns about population decline today are economists (e.g., (Fernandez-Villaverde, 2025; Spears & Geruso, 2025).

Some also argue that immigration will reduce the impacts of low fertility. Among countries that are able and willing to allow substantial immigration, migration can slow or even prevent population decline, although it is less capable of preventing population aging (Bagavos, 2022; Craveiro et al., 2019; Parr, 2023). However, immigration is likely not a long-term solution to the problem of population decline either, as fertility is falling across the world. Immigration does not add to the planetary stock of people, suggesting that while it might help some countries for some period of time, it will hurt others that lose population (Evans, 2024; Fernandez-Villaverde, 2024; Kumar, 2024). In addition, in countries with robust welfare systems, immigrants’ contributions to a country’s fiscal health may be minimal, especially as they themselves age (Fernandez-Villaverde, 2025; Smith, 2026).

Others argue that a variety of policy interventions, such as changing the way Social Security is funded, increasing automation, or more fully funding childcare, can either reduce the impact of fertility decline or largely stem it (Root et al., 2025; Skirbekk, 2023). However, each of these approaches has important drawbacks that I address in a later section.

## The moral framing of low fertility

Just as there were a mix of pragmatic and ideological concerns about rapid population growth, there is a similar mix in conversations of low fertility and future population decline, which is occurring both among demographers and the broader public. Discussions today are perhaps even more fraught due to high and increasingly levels of political polarization (Iyengar et al., 2019).

I propose a conceptual framework that distinguishes between two broad moral orientations—Social Justice and Social Order—that structure how individuals interpret the causes and consequences of low fertility (Iceland et al., 2023). The framework is useful for not just documenting that people disagree, but why they do so, and along what dimensions. It should be said that Social Justice and Social Order are not mutually exclusive categories, but ideal types that help clarify patterns in how low fertility is understood and debated.

These views are drawn in large part from Moral Foundations Theory (MFT) (Graham et al., 2011, 2013; Haidt et al., 2009). MFT proposes that people have moral intuitions—gut-level reactions—about how the world should work and what makes for a good, functioning society. There are two broad types of moral intuitions: individualizing—or what can be termed *Social Justice*—intuitions emphasize avoiding harm and ensuring fair treatment of others, especially the vulnerable; while binding—*Social Order*—intuitions focus on preserving the order, stability, and cohesion of social groups (Iceland et al., 2023; Silver et al., 2023). These intuitions emerged in humans in response to adaptive challenges in our evolutionary past (Graham et al., 2013; Haidt et al., 2009).

Specifically, Social Justice intuitions have evolutionary roots in parental care and kin altruism. Humans are unique in how long infants remain dependent on their parents. Without an innate drive to provide care, humans would not have survived. Social Order intuitions emerged to enhance cohesion and cooperation—both essential for communities to flourish. In pre-modern times, survival depended on cooperation to hunt, defend territory, and share resources. Without strong group bonds and trusted leadership, our ancestors would not have been able to survive hardship and external threats to their safety. Individuals and groups differ in the strength of these intuitions in part due to heritable variation but also largely because of social learning. Parents impart values to their children, and cultures vary in the extent to which they promote different cultural norms (Graham et al., 2009, 2013). MFT generally holds that moral intuitions generally precede conscious reasoning and shape people’s policy preferences (Haidt, 2012). Policy positions or group interests are then often subsequently rationalized in moral terms.

The features of each of these moral viewpoints that are most important to understanding why people disagree about fertility decline is their different views on: (1) which unit (individual vs. group) is the focal concern of decision-making, and (2) the appropriate pace of social change. Social Justice focuses on reducing harm inflicted on vulnerable, traditionally marginalized individuals, while Social Order is most concerned with stability, order, and group cohesion, which is deemed important for collective well-being. Social Justice is comfortable with sweeping, structural change, given that existing structures often embody unfair practices, while Social Order tends to favor incremental change, fearing that the instability that might result from far-reaching change may do more harm than good (Iceland et al., 2023).

### The link between moral frames and fertility trends

There are reasons to believe that over the past several decades, trends in fertility have been increasingly framed from a *Social Justice* perspective not only among left-leaning commentators but also sociologists and demographers. A number of scholars have documented the strong influence of social justice thinking and activism with sociology (Iceland, 2025; Iceland & Silver, 2024; Rubin, 2026; Smith, 2014; Turner, 2019). This likely reflects the increasing ratio of self-identified Democrats to Republicans among faculty members (Langbert, 2018; Magness & Waugh, 2023; Martin, 2016). Empirical work has also documented the increasing focus on inequality in both sociological and demographic research—inequality being a concern central to the Social Justice moral orientation (Iceland et al., forthcoming; Keskinturk, 2023; Moody & Light, 2006).

Indeed, the promotion of fertility *decline* has often been framed from a *Social Justice* perspective, as the decline reflects progress toward women’s empowerment, greater control over reproduction, and more opportunities for self-determination. In this framing, the central moral concern is the individual—specifically women, a historically marginalized group under patriarchal norms in many traditional societies. In this view, concerns about population decline risk veering into reactionary territory, including nostalgia for patriarchy or anxiety about national decline. The focus on women’s empowerment thus calls for far-reaching structural changes in traditional societies.

In linking fertility decline and Social Justice concerns decades ago, Lesthaeghe (1983: 411) similarly argued, “A fertility decline is in essence part of a broader emancipation process. More specifically, the demographic regulatory mechanisms, upheld by the accompanying communal or family authority and exchange patterns, give way to the principle of individual freedom of choice, thereby allowing an extension of the domain of economic rationality to the phenomenon of reproduction.”

Conversely, exemplifying the view that pronatalism is a vehicle for promoting reactionary causes in the broader public discourse, Nandita Bajaj (2025), writing in the magazine *Ms.*, argues that “Even if contemporary pronatalism and depopulation alarmism are couched in terms designed to sound agreeable to the left, that doesn’t change the fact that they are linked to a host of alarming ideologies: far-right extremism, autocratic regimes, the Great Replacement conspiracy theory, eugenics, racism and sexism…Any progressivism or feminism worthy of the name is about expanding rights and choices. If we truly believe in those fundamental pillars, we cannot put women’s reproductive choices on the table as currency to be exchanged for government kickbacks.”

Similarly, the increased focus on low fertility is seen by many as an attempt to scapegoat people—mainly women—for issues that could be addressed in other ways, such as by raising taxes to pay for fiscal deficits incurred by an aging population (Booth, 2024). In support of this view, some efforts to increase fertility have indeed downgraded the importance of women’s empowerment. In China in 2023, for instance, government officials eliminated gender inequality as a priority and advised women “to establish a correct outlook on marriage and love, childbirth, and family” (Booth, 2024), and essentially promoting traditional gender roles (Stevenson, 2023).

Skepticism of the motivations among pronatalist proponents extends to academic commentators, who note that concerns about population decline are more prevalent among conservatives than liberals (Cohen, 2025), argue that pronatalist proposals often overlook tradeoffs or the well-being of the children the movement may produce (Folbre, 2025), or contend that they reflect a moral panic that scapegoats women and advances populist or ethnonationalist agendas (Gietel-Basten, 2023). While each of these critiques draw on supporting evidence, they do not establish that all advocates of higher fertility are ideologically motivated, nor that low fertility is not a serious problem. Depopulation linked to sustained low fertility can carry real consequences for labor markets, public finances, and communities—consequences that warrant serious attention even among those skeptical of how the issue is sometimes framed.

That said, low fertility and population decline *do* pose a threat to moral concerns of those concerned with Social Order. From this perspective, low fertility is evidence of both demographic decline and social decay. It signals weakening families, declining religiosity, and an erosion of collective purpose. Parenthood and marriage are seen as critical social institutions, and so their decline is seen as an indication of a weakening of important cultural norms that sustain healthy communities. In this way, it highlights the well-being and cohesion of groups rather than just the care of vulnerable individuals as in the Social Justice perspective. Social Order is also wary of the sweeping social changes in family living arrangements underway that are of much less concern from the Social Justice perspective.

There are numerous examples of the Social Order viewpoint on fertility decline. Writing in *National Affairs*, Abby McCloskey (2025) argues that “America’s fertility rate has plummeted. In response, politicians are floating a plethora of policies designed to encourage Americans to have more babies. These “pro-natalist” efforts, however, are taking place within a larger broken culture of fertility in America, which includes inconsistent understandings of life, callous policy treatment of mothers, and preventable difficulties in birth. Addressing our crisis of fertility must go beyond policy and nourish a new culture of family and fertility from the bottom up.”

From the Social Order perspective, those who seem unconcerned with fertility declines, or who seemingly denigrate motherhood, are ideologically motivated and in denial about the problems posed by low fertility. In her criticism of books and magazine articles that highlight the dangers of pregnancy, often in reaction to the pronatalism of those on the right, Kara Kennedy (2025) argues that “We live in a country where birth rates are plunging, even though the majority of young Americans still say they want children. Yet Democrats seem incapable of presenting a positive vision of pregnancy, childbirth, or parenting”.

This anti-natalism has been observed by those on the left as well. As Victor Kumar, a professor of philosophy, argues:

The left has responded in a manner typical of modern political culture — through negative partisanship. They think: “if these people believe population decline is a problem, then it must not be one; if these are the solutions, the problem must be imaginary.” Of course, this reasoning is specious. A proposed cure may be awful while the disease is real. The left will accept that population decline is a genuine problem only if we can develop a “progressive pronatalism” that offers better solutions while explicitly rejecting gender traditionalism and nativism. (Pethokoukis, 2025)

Kumar empathizes with the progressive reaction against pronatalism, as he agrees that many proposed solutions are reactionary—such as pressuring women to abandon careers for full-time parenting— and because prominent voices sounding the alarm, like Elon Musk and JD Vance, are political opponents of progressives. Yet the merits of an argument cannot be assessed solely by the motivations (or character) of those who make it, and sustained fertility decline, in his view, likely poses real challenges regardless of who raises the concern (Kumar, 2024).

In short, many people are skeptical of good-faith arguments about population decline because some advocates of higher fertility are, in fact, motivated by moral commitments they find objectionable. This mirrors earlier suspicions of the coalition that promoted fertility reduction in the 20th century, which was itself driven by a mix of motivations—including eugenic ones now widely regarded as morally indefensible. In both cases, the ideological leanings of some advocates have made it harder to engage the underlying demographic questions on their merits.

## Empirical analysis of the link between moral frames and fertility in demographic research

While the previous section outlined the theoretical link between moral intuitions and the framing of fertility issues—and reasons why we might expect academic work to be more heavily influenced by the Social Justice moral frame—here this is put to an empirical test. The analysis is based on data obtained through Scopus API—an abstract and citation database—using the serial numbers (ISSNs) of eight prominent, English-language demography journals through July 2025, with the starting year in parentheses: *Demographic Research* (1999), *Demography* (1964), *European Journal of Population* (1985), *Genus* (1970), *Population* (2001), *Population and Development Review* (1976), *Population Research and Policy Review* (1982), *and Population Studies* (1947). While two of these journals have older issues (*Genus* and *Population*), the database only includes English languages volumes available in Scopus. The search returned 12,044 publications including 11,309 research articles plus many reviews, notes, editorials, and errata. The analysis begins with the 11,309 research articles, since these are the main source of new academic work in these journals. To avoid research articles with limited semantic information, all articles that had less than 20 words in their titles and abstracts combined were omitted. These articles were either misclassified by Scopus as research articles or had missing titles or abstracts, which reduced the sample to 10,411 articles. Finally, the analysis was run on the 3,249 papers from these whose titles and abstracts included the keywords *fertility* or *birth rate(s)*.

To analyze the application of alternative moral frames in demographic research, Deepseek V4 Flash was used, which is one of the most capable models available at this time. It was prompted with: “You are an expert social science text coder specializing in framing analysis. Classify the Target Text ONLY on what it explicitly states. Do not infer beyond the snippet. Absence of framing language means Neither/Unclear, not a hidden frame.” The moral frames were defined as follows:

*Social Justice*: frames fertility in terms of fairness, rights, or autonomy, OR harm to vulnerable / traditionally marginalized individuals (e.g. women, low-income families, minorities). Typical markers: equality, equity, autonomy, choice, rights, coercion, patriarchy, discrimination, burden of care when tied to a fairness/rights claim. *Social Order*: frames fertility in terms of collective continuity, stability, or institutional order (nation, population, tradition, family as institution). Typical markers: stability, continuity, tradition, collapse, crisis, extinction, threat to nation/population when tied to a claim about collective well-being or decline-as-problem. The appendix includes additional information about edge rules and provides examples of how different cases were coded.

Papers were coded into three categories: Social Justice, Social Order, and neither (there were no papers that used both frames). Definitions for each were provided in the prompt. The model was iteratively tested on randomly drawn samples and exclusion criteria were added (also included in the prompt) to make its predictions more conservative in assigning a publication to either Social Justice or Social Order (rather than neither).

### Results

Of the 3,249 papers with included fertility or birth rates in the titles or abstracts, 92.0% did not have an explicit frame, 6.7% had a Social Justice frame, and 1.4% had a Social Order frame. Thus, a vast majority of paper did not have an explicit frame in its title or abstract, but among those that did, most had a Social Justice frame (83%) rather than a Social Order one (17%).

Among those that adopted one of the two frames, I also examined the share of papers adopting each by topic (see Table 1). Among papers examining only fertility increases, 83% adopted Social Justice framing, often with a focus on reducing fertility by increasing schooling and women’s autonomy. Among papers that focused on low/declining fertility, most papers (66%) still framed it with a Social Justice orientation, but there were relatively more papers (as compared to fertility-increase papers) that framed it as a Social Order concern (34%). Those from a Social Justice perspective often proscribed gender equity/work-family balance as the remedy for low fertility. There were few papers that examined both high and low fertility (16 in all), and these were roughly balanced between Social Justice (56%) and Social Order (44%). Finally, among papers that did not focus on either rising or falling fertility, all were framed with a Social Justice orientation—these papers often discussed issues such as sex selection, reproductive autonomy, contraceptive access, and gender discrimination.

**Table 1:** Number and percent of papers with a social justice and social order orientation, by topic

Topic	Social justice (217 papers)	Social order (44 papers)
Low/falling fertility	59 (66%)	31 (34%)
High/rising fertility	29 (83%)	6 (17%)
Both	9 (56%)	7 (44%)
Level fertility	120 (100%)	0 (0%)

**Fig. 1:**
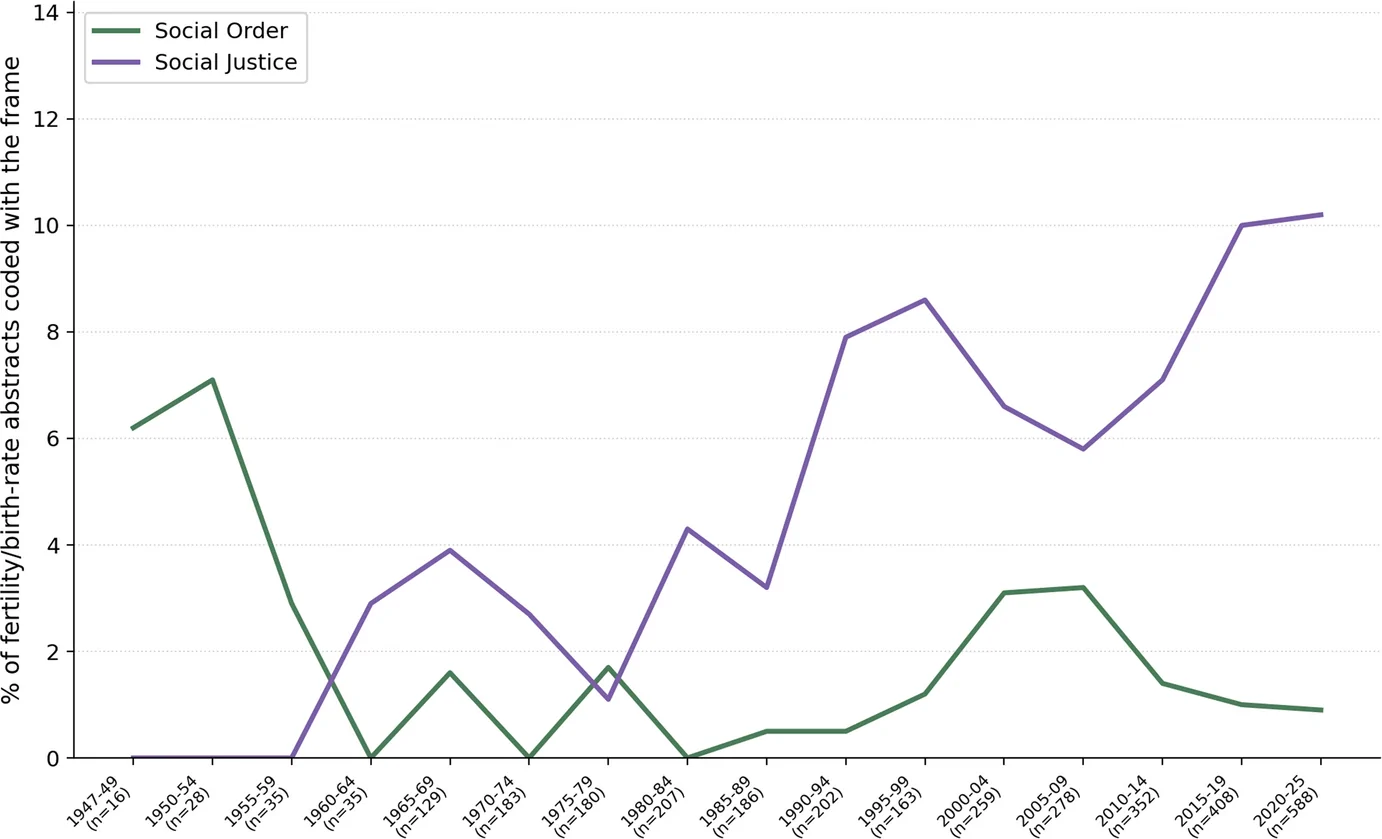
Trends in the Social Justice and Social Order framing of papers on fertility

Figure 1 shows the change in the Social Justice and Social Order framing of fertility papers from 1940 to 2025. As noted in the overall statistics on moral framing, most papers did not have a Social Justice or Social Order framing, at least in the title and abstracts. Among those that did, in the 1940s into the 1950s, papers on fertility tended to use Social Order framing of fertility issues, likely focusing on the need for fertility control in developing countries to spur economic development and modernization (Coale & Hoover, 1958). However, beginning in the 1960s, Social Justice framing increased, before accelerating in the 1980s and reaching new peaks in the 2010s and early 2020s. This is consistent with previous research showing an increasing focus on inequality in demographic research more generally (Iceland et al., forthcoming).

## Policy responses, evidence on effectiveness, and moral frames

Responses to population decline generally fall into four categories: technical adaptation; social change that increases work-family balance and gender equality; economic adaptations and incentives that address fiscal problems and promote increases in fertility, respectively; and cultural change that promotes fertility. The second of these is aligned with the Social Justice perspective while the last is most consonant with Social Order. The other two have elements that may appeal to each perspective, as well as other elements that raise concerns (see Table 2).

**Table 2:** The acceptability of different policy approaches to each moral orientation

Policy response	Moral orientation	
	Social justice	Social order
Technical adaptation	Neutral—with concerns	Neutral—with concerns
Promote work family balance and gender equality	Preferred	Concerns
Economic adaptations and incentives	Neutral	Neutral
Cultural change to promote fertility	Opposed	Preferred

Technical adaptation seeks to offset demographic decline through innovation, such as automation and robotics. Automation can replace human labor where it is scarce, which may be exacerbated by population decline (Skirbekk, 2023). Cross-national evidence indicates that countries with slower population growth and population aging are more likely to adopt automation to cope with demographic change (Abeliansky and Prettner 2023; Acemoglu and Restrepo 2022a; Stahler 2021). Indeed, some have voiced concern that AI-driven automation will lead to job losses in various industries (Nigar et al., 2025).

However, while automation may help in in places where human labor is scarce, it is unlikely to solve all problems. First, it won’t fix the fiscal challenge of an aging population, and population decline may in fact stifle innovation over the long run (Jones, 2022). In addition, while AI could reduce employment in some industries, there are also currently many jobs that could not easily be done through automation, such as child and elder care, where there are current labor shortages (Jones & Dolsten, 2024). It is thus far from clear whether automation alone will solve many of the challenges posed by sustained low fertility.

Support for automation doesn’t cut clearly across moral viewpoints. From the Social Justice perspective, automation is not inherently objectionable, but there are concerns that it could adversely affect low-skill and unionized workers, and that gains may be captured disproportionately by corporations and wealthy individuals (Acemoglu and Restrepo 2022b; Stahler 2021). Thus, objections stem from concerns that it will harm vulnerable populations and exacerbate inequality. From the Social Order perspective, automation is likewise not inherently objectionable, but similar concerns arise about labor displacement, the declining centrality of work, and the downstream effects on family formation and community cohesion (Cass, 2018). The two perspectives thus converge less on enthusiasm for automation than on a shared wariness about how it is implemented and who might bear the costs, such as vulnerable populations or families and the communities.

The social change approach to moderating fertility decline, which emphasizes work-family balance and gender equality, holds that fertility can rise only when parenthood becomes more compatible with modern life. Thus, family policies should focus on lowering the costs of having children, and especially among women, who currently bear the larger burden of caring for children. Specific policies include providing subsidized or free childcare, reducing the cost of childcare in other ways, such as less regulation, expanding parental leave, increasing the fathers’ share of childcare responsibilities coupled with a change in social norms that facilitates gender egalitarianism, and flexible labor markets that facilitate the balance between work and family obligations (Doepke et al., 2023; Esping-Andersen & Billari, 2015; Flowers et al., 2024; Kumar, 2024; McDonald, 2000; Piano & Stone, 2025). These can lower the cost of caring for children on women, and thus increase childbearing among couples where women might otherwise want fewer children than men (Doepke & Kindermann, 2019). These could also help people achieve their fertility intentions, as most data suggest that people are not having as many children as they intend (Morgan & Taylor, 2006). Nordic models exemplify the social change approach with strong social welfare programs and high levels of gender egalitarianism.

In a review of studies that examined the effects of policies such as childcare and parental leave on fertility, Hart et al. (2024) concluded that these policies have a positive, though modest effect on fertility (see also Kuhn et al., 2026). These types of policies alone, however, appear insufficient to restore fertility to near replacement levels. Despite a partial rebound in the 2000s, Sweden, Denmark, Norway, and Finland all recorded all-time low TFRs in 2023, ranging from a low of 1.3 (Finland) to a high of 1.5 (Denmark) (World Bank Group, 2026). This is lower than the 1.6 TFR in the U.S., which is less egalitarian and has weaker supports for parents (Stone, 2018). The recent declines in fertility could reflect that although egalitarianism might reduce the costs of having children for women, fertility intentions are generally low among individuals who have gender egalitarian attitudes (Begall & Hiekel, 2025). This suggests that structural supports and egalitarian social change alone likely cannot overcome low fertility without even deeper institutional changes and shifts in values (Kearney & Levine, 2026).

Gender egalitarianism is one of the cornerstones of Social Justice, so this approach to addressing fertility decline is very consonant with this moral framework, as is its call for significant social change in places with significant gender gaps in the paid labor market and home production. Gender egalitarianism is not a key feature of the Social Order perspective. While there are few Social Order commentators in Western countries who would explicitly promote gender inequality, they generally endorse having a parent—usually the mother—stay at home to raise children, especially while the children are young. They often argue that our current culture discourages and demeans stay-at-home mothers, even though many women would otherwise like to provide this care for their children (Greco 2023a, 2023b; Wilcox & Wang, 2022). Thus, the social change approach to increasing fertility has stronger support among proponents of Social Justice than those of Social Order, who are wary of extensive social changes.

The third approach to increasing fertility is through economic adaptations and incentives, which include: (a) controlling spending on programs that support aging populations to address fiscal challenges, and (b) providing economic incentives—such as cash transfers, tax benefits, or child allowances—that offer financial compensation for childbearing to increase fertility.

The fiscal challenges of supporting an aging population are widely recognized. These challenges can likely be met by raising the age of retirement and adjusting how Social Security is funded, such as by increasing payroll taxes (Root et al., 2025; Skirbekk, 2023). Healthcare spending can be curbed by, among other practices, drug price negotiation, reducing waste and overpayments, raising the eligibility age for Medicare, rationing care, greater cost sharing, or increasing taxes (Committee for a Responsible Federal Budget, 2025; Cubanski et al., 2019; Keyser & Ducas, 2024, 2024; Moffitt, 2016). However, many of these proposals would likely be unpopular. Medicare and Social Security are among the most popular government programs in the U.S. (Sanders, 2023). The elderly also wield considerable political power, in part because of higher voter turnout than among younger groups, making policies contrary to their interests politically contentious and difficult to enact (Barilari et al., 2025).

Many countries have introduced economic incentives to increase fertility. In addition to subsidized day care and extending paid parental leave (discussed above), these have included cash payments at birth, tax exemptions and credits, and child allowances and monthly payments.^1^ Economic incentives to increase fertility, which have been implemented in a variety of settings, have been found to have a positive, though modest effects on fertility (Hart et al., 2024). There is also some evidence suggesting that the increases in fertility they produce may end up being temporary (Gauthier & Gietel-Basten, 2025). For instance, since 2010, Hungary has implemented a variety of policies, including zero-interest loans and debt forgiveness for couples with children, personal income tax relief for mothers, and housing support for married couples. The TFR rose from 1.2 in 2011 to 1.6 in 2021, before falling back to 1.4 in 2024. Other countries with pronatalist policies, such as Belarus, Bulgaria, Estonia, Japan, Singapore, and South Korea have also achieved little success in boosting fertility for a sustained period of time (Brown Calder, 2025).

The evidence also suggests that low fertility is not just about affordability. Poorer countries have higher birth rates on average than richer ones, and, historically, fertility has generally declined in countries as standards of living have risen (Spears & Geruso, 2025). Rather, lower fertility may result in part from greater *opportunity costs* of having children. In decades and centuries past, there was less to lose by spending time on children. Today, in places with high standards of living, the opportunity costs of parenting today include forgone vacations, restaurant meals, streaming movies, being on social media, or spending quality time with a partner, and especially for women, more income that might allow them to better afford these things (Evans, 2024 ; Kearney et al., 2022; Spears & Geruso, 2025). The implication is that would likely take a lot more money to increase fertility than an incrementally larger child tax credit. Currently, aid for families is a fraction of that devoted to programs for the elderly, and that would likely need to change dramatically to make a difference, and so far we do not have evidence that it would (Santa, 2026).

As with automation, neither the Social Justice nor Social Order perspective is inherently opposed to economic policy interventions aimed at addressing fiscal challenges of an aging population and other aimed at stemming fertility decline, though they tend to favor different approaches that are more consonant with their moral concerns. For example, the Social Justice perspective is more likely to support expanded childcare subsidies and paid parental leave, as these ease the burden on working women, advance egalitarian goals, and help families meet basic needs (Gauthier & Gietel-Basten, 2025). They are also willing to raise taxes to fund them. People from the Social Order perspective, while generally skeptical of expansive government programs, tend to view low fertility as serious enough to warrant significant public spending, emphasizing direct cash transfers an expanded child tax credit rather than gender-egalitarian workplace policies. These approaches are intended to promote family formation and childrearing in ways that are not incompatible with more traditional family roles (Wilcox, 2026; Wilcox & Wang, 2022).

Finally, there’s the cultural renewal approach to stemming population decline, which seeks to revalorize family, faith, or civic duty to increase fertility. Some countries, such as Poland, Russia, and Hungary, fertility promotion has been linked to an agenda of promoting conservative family and gender norms (Cook et al., 2023; Gauthier & Gietel-Basten, 2025). Some proponents of cultural change, however, see the limitations of government action alone, as cultural change needs to be nourished by grassroots efforts. As a Heritage Foundation report on the need to strengthen families notes (Severino et al., 2026): “The recommendations in this report are downstream from the transformative work that must be done in other parts of the culture. Families, communities, religious institutions, and other civic institutions must enrich the soil and plant the seeds of marriage and family. The government’s primary role is to clear the weeds and prevent its policies and programs from poisoning the ground.”

So far, family policies and traditionalist approaches—such as those mentioned above that were implemented in a few Eastern European countries—have had limited effects on either reversing demographic decline or changing gender norms (Cook et al., 2023). Whether cultural changes will occur that are more conducive to higher fertility is hard to predict, but at this time they are not occurring, as evidenced by continuing worldwide declines in fertility.

Those from the Social Justice perspective oppose any approach that promotes the cultural retrenchment described above. It is precisely these cultural proposals that make many progressive-minded individuals instinctively dismissive of concerns about low fertility, and suspicious of pronatalist policies more generally. The cultural changes invoked either implicitly or explicitly proscribe more traditional gender norms that are associated with women’s disadvantage in the paid labor force. It is thus deeply at odds with the gender egalitarianism promoted by progressive commentators (e.g., Bajaj 2025; Gietel-Basten, 2023). Conversely, those from the Social Order perspective generally support cultural change oriented toward the renewal of traditional family formation, viewing such changes as a means not only to increase fertility but also to strengthen families and community life (McCloskey, 2025; Wilcox & Wang, 2022).

## Discussion

What a difference a century makes. The Population Association of America was founded 100 years ago when population growth began to gain attention as a serious social problem. This concern culminated in the implementation of population control programs in the decades after World War II in a wide array of countries in the developing world. Although rapid population growth posed real challenges, efforts to reduce it arose from a coalition of people motivated by a range of ideological and moral commitments. Over time, some of these, such as eugenics, receded, while others, such as women’s empowerment, were foregrounded by policy makers and demographers.

Today, low fertility is emerging as critical issue. Persistent low fertility, combined with low mortality, leads to an aging population. This strains safety nets that provide generous benefits to the elderly, such as through income assistance (e.g., Social Security) and medical care (e.g., Medicare). A declining population resulting from sustained low fertility could slow economic growth due to declines in aggregate demand, population aging, and lower levels of innovation. Low fertility produces smaller kin groups and kin support—both critical for physical and emotional support. At the individual level, low fertility sometimes also reflects the inability of people to meet intended fertility goals.

Those who draw attention to the problem of low fertility are motivated by a mix of practical concerns and ideological commitments. There are real substantive disagreements about whether low fertility will really have a negative impact on society. For example, some commentators are wary of long-term forecasts, others hold that the challenges of low fertility can be met with reasonable policy responses, and yet others believe that immigration can relieve the impact of low fertility in many countries.

However, I argue that differing viewpoints rooted in Social Justice and Social Order moral concerns also contribute to differences in opinion on fertility issues. Social Justice emphasizes reducing harm inflicted on individuals who are members of historically marginalized groups, including women. Social Order emphasizes preserving the order, stability, and cohesion of social groups. By clarifying the moral frameworks underlying some of the disagreements about population decline, this paper aims to provide a foundation for future empirical work and for more productive engagement across ideological and moral divides.

At a time when Social Justice concerns had been foregrounded in academic institutions and many international organizations, fertility reduction was justified as a vehicle for women’s empowerment, as highlighted in the 1994 Cairo conference on population and development. But now that sustained fertility decline is a potential problem, there is genuine tension between Social Justice concerns and the means to increase fertility. First and foremost, there is the concern that the burden of increasing fertility will disproportionately fall on women—a fair concern indeed given that women have historically borne this burden.

To examine the extent to which moral concerns help shape debates about fertility, I conducted an empirical analysis of demographic research on fertility in eight leading demography journals published from 1947 to 2025. Results indicated that while most article abstracts did not have a particular moral frame, among those that did, most (83%) had a Social Justice frame rather than a Social Order one (17%). The prominence of Social Justice framing also increased over time.

This is consistent with Cohen’s (2025) analysis showing that concerns about falling birth rates skew conservative among the American public. Similarly, many demographers also have legitimate concerns that some pronatalists seek to restore traditional gender norms. Thus, when demographers do engage, they tend frame it as a relatively minor problem or promote gender egalitarianism as the main remedy (Guzzo, 2026; PBS News, 2025; Tavernise & Adelson, 2026). This may reflect the broader ideological composition of academia, where scholars disproportionately hold progressive views and are more likely to adopt Social Justice oriented frameworks (Langbert, 2018; Magness & Waugh, 2023). Such orientations shape research agendas, directing attention toward issues such as inequality (Iceland, 2025; Jussim et al., 2024), while placing less emphasis on concerns more closely with the Social Order perspective, including low fertility.

Finally, I reviewed four general types of policy responses to low fertility that vary in the extent to which they appeal to people with Social Justice vs. Social Order concerns: (1) efforts to promote work-family balance and gender equality, (2) technical adaptations (such as automation), (3) economic adaptations and incentives (such as increasing child allowances and reducing spending on the elderly), and (4) cultural change to promote fertility. The first of these, the focus on women’s empowerment as a vehicle for fertility decline, reflects a Social Justice approach to reducing fertility, as it emphasizes equality and the protection of women’s rights in traditional, patriarchal societies, and advocates for extensive social changes along these lines. Conversely, the fourth of these, which emphasizes the importance of strengthening families and communities, reflects a Social Order approach to reducing fertility. The other two (technical and economic adaptations) are not clearly aligned with one orientation or another, though they have elements that are more and less appealing to each moral orientation. So far, no single approach appears to have more than a modest impact on fertility, though we may find that in combination, they may yield larger effects over time and reduce the likelihood and pace of population decline.

Overall, this review suggests that unlike during the period of rapid population growth, when demographers were deeply involved in efforts to reduce fertility, demographers today often appear more reticent to highlight the potential problem of low fertility (Evans, 2024; Spears & Geruso, 2025; Tavernise & Adelson, 2026). Put another way, it’s not that demographers aren’t studying fertility decline—low fertility is featured in many demographic research articles—it’s just less likely to be featured as a *serious social problem* requiring a significant and wide array of policy interventions that may need to extend beyond gender egalitarianism. The reticence among some commentators undoubtedly reflects honest scientific uncertainty about the pace and effect of low fertility, but among others it likely also reflects differences in moral mindsets.

This does not mean that demographers, or social scientists more broadly, should abandon their moral commitments. Indeed, the Social Order perspective has its own blind spots, such as underplaying the real costs that some pronatalist policies impose on women. Rather, it suggests the importance among scholars of recognizing that their own commitments are not universally shared. Greater engagement with the challenges posed by low fertility, and with the full range of perspectives on how to address it, could help foster more constructive dialogue and more broadly acceptable policy responses. This may include consideration of approaches that are not fully aligned with Social Justice priorities but are not necessarily incompatible with them, such as technological adaptation or expanded economic incentives for increased childbearing.

For example, technological adaption might play an important role in mitigating the negative effects of depopulation, even if existing technologies are currently insufficient. During the earlier period of rapid population growth, when concerns about resource scarcity and sustainability were common, unforeseen technological innovations helped address these challenges, such as through gains in agricultural productivity and the development of cleaner energy sources. Similarly, emerging technologies, including advances in artificial intelligence and robotics, could increase productivity and help offset labor shortages, with the potential to generate the tax revenues from this productivity growth needed to sustain welfare states (Congressional Budget Office, 2024).

One demographic goal that could draw support from both moral perspectives is helping women achieve their desired level of fertility, as women in Europe, the United States, and many other countries are currently having fewer children than they originally intended (Beaujouan & Berghammer, 2019; Berrington & Pattaro, 2014; Harknett & Hartnett, 2014; Lazzari & Beaujouan, 2026). The preferred means to do so, however, may differ somewhat across the moral orientations, but it could, for instance, include a mix of policies centered both around gender egalitarianism and economic incentives that provide more support for families wishing to have more children, such as more generous child allowances.

In conclusion, demography should remain a discipline grounded in empirical rigor while maintaining awareness of its normative concerns. Just as population control efforts were, over time, guided by an evolving mix of moral commitments, so too will responses to population decline be shaped by the values that guide both scholars and policymakers. The task for demographers is not to eliminate these moral dimensions, but to engage with moral issues more openly to better understand and more effectively respond to the demographic challenges ahead.

## Data Availability

Data are available upon request.

## Appendix: edge rules for coding and coding examples

All articles were coded as having one of the following three frames: Social Justice, Social Order, or neither/unclear (no articles used both Social Order and Social Justice frames).

### Edge rules

1. Measurement language ALONE is not Social Order: decline, tempo, replacement, momentum, TFR, parity, postponement count as Social Order ONLY if the text frames them as a threat to collective continuity—not when used purely descriptively / methodologically.
2. Policy/instrument mentions ALONE are not Social Justice: childcare, parental leave, contraception, employment, housework count as Social Justice ONLY if tied to fairness, autonomy, rights, or harm-to-vulnerable—not when studied as neutral correlates.
3. Country cases and methods with no evaluative claim -> Neither/Unclear.
4. Stance:

- Reported frames do not count: a frame merely mentioned as widespread ("concerns are widespread"), posed as an open question, or quoted from others without endorsement -> Neither/Unclear.
- Substantively rejected claims do not count: a frame raised only to be refuted on factual grounds ("this argument is a fallacy") -> Neither/Unclear.
- But value-defense and coercion-critique do count: (i) rejecting the misuse or instrumentalization of a value counts as endorsing that value. For example, criticizing the use of gender equality for demographic ends because it "preserves inequality" or "narrows" equality IS a Social Justice stance, grounded in fairness;^2^ (ii) characterizing a fertility policy with coercion/cruelty language ("draconian", "coercive", "forced", "harshly enforced", "nightmares") counts as Social Justice framing (harm/autonomy), even inside a technical narrative. Mere topical mention of a policy ("population control", "one-child policy") with no evaluation still counts as neither Social Order or Social Justice.

#### Distinguishing test for rejections

does the rejection of claim itself invoke fairness/rights/harm (code Social Justice) or continuity/stability/threat (code Social Order)? If it invokes neither, code Neither/Unclear.

### The LLM was prompted to produce the following output

#### Output

Respond with ONLY a valid JSON (output format) object with exactly these keys:

- Orientation: one of Social Justice, Social Order, Both, Neither/Unclear
- confidence: integer 1-5 (1 = guess, 5 = explicit framing language)
- quote: String (a short VERBATIM span from the text supporting the call; empty string if Neither/Unclear; no paraphrase, no invented words)
- rationale: String (1-2 sentences linking the quote to the label)

### Coding examples and output

- Example 1. Text: "The demographic desirability of low birth rates achieved at the cost of reducing female births needs to be examined."
- JSON: {"orientation": "Social Justice", "confidence": 4, "quote": "at the cost of reducing female births", "rationale": "Frames low fertility as harm to a vulnerable group (females) via sex-selective practices."}
- Example 2. Text: "Critics touted empire as a solution to the damaging effects of low birth rates and other degenerative pathologies."
- JSON: {"orientation": "Social Order", "confidence": 5, "quote": "solution to the damaging effects of low birth rates", "rationale": "Frames low fertility as macro-level degeneration threatening collective strength, with empire as restoration of order."}
- Example 3. Text: "Most of the demographic literature considers the aging of the society as a socioeconomic disaster, threatening the whole social structure. This argument is a fallacy, because productivity is much more important than numbers of employed persons."
- JSON: {"orientation": "Neither/Unclear", "confidence": 4, "quote": "", "rationale": "The threat language is reported from other literature and refuted on substantive grounds (''this argument is a fallacy''); the rejection invokes no fairness or continuity value, so rule 4(b) applies."}Example 4. Text: "This paper argues against pushing for gender equality aggressively in order to raise fertility. It rejects this pursuit, maintaining that this method preserves inequality, and warns against using gender equality for fertility purposes, stating that this narrows the realm of gender equality."
- JSON: {"orientation": "Social Justice", "confidence": 4, "quote": "this method preserves inequality", "rationale": "The text rejects instrumentalizing equality for demographic ends on fairness grounds (''preserves inequality'', ''narrows the realm''); defending the value against misuse is an endorsed SJ stance under rule 4(c)."}"""

## Footnotes

1 Taking another tack, Mahler et al. (2025) argue that high parental investments in children—a response to comparisons and competition—contribute to lower fertility, so one way to reduce this investment arms race is to tax or regulate some private college and universities or reform college admissions to stimulate fertility.
